# Composition and Codon Usage Pattern Results in Divergence of the *Zinc Binuclear Cluster* (*Zn(II)_2_Cys_6_*) Sequences among Ascomycetes Plant Pathogenic Fungi

**DOI:** 10.3390/jof8111134

**Published:** 2022-10-27

**Authors:** Shilpi Bansal, Mallana Gowdra Mallikarjuna, Alexander Balamurugan, S. Chandra Nayaka, Ganesan Prakash

**Affiliations:** 1 Division of Plant Pathology, ICAR—Indian Agricultural Research Institute, New Delhi 110012, India; 2 Division of Genetics, ICAR—Indian Agricultural Research Institute, New Delhi 110012, India; 3 Department of Studies in Applied Botany and Biotechnology, University of Mysore, Mysore 570005, India

**Keywords:** Ascomycetes, codon usage bias, fungi, compositional constraints, host–pathogen interaction, natural selection, zinc binuclear cluster family

## Abstract

Zinc binuclear cluster proteins (ZBC; Zn(II)_2_Cys_6_) are unique to the fungi kingdom and associated with a series of functions, *viz.*, the utilization of macromolecules, stress tolerance, and most importantly, host–pathogen interactions by imparting virulence to the pathogen. Codon usage bias (CUB) is the phenomenon of using synonymous codons in a non-uniform fashion during the translation event, which has arisen because of interactions among evolutionary forces. The *Zn(II)_2_Cys_6_* coding sequences from nine Ascomycetes plant pathogenic species and model system yeast were analysed for compositional and codon usage bias patterns. The clustering analysis diverged the Ascomycetes fungi into two clusters. The nucleotide compositional and relative synonymous codon usage (RSCU) analysis indicated GC biasness toward Ascomycetes fungi compared with the model system *S. cerevisiae*, which tends to be AT-rich. Further, plant pathogenic Ascomycetes fungi belonging to cluster-2 showed a higher number of GC-rich high-frequency codons than cluster-1 and was exclusively AT-rich in *S. cerevisiae*. The current investigation also showed the mutual effect of the two evolutionary forces, *viz*. natural selection and compositional constraints, on the CUB of *Zn(II)_2_Cys_6_* genes. The perseverance of GC-rich codons of *Zn(II)_2_Cys_6_* in Ascomycetes could facilitate the invasion process. The findings of the current investigation show the role of CUB and nucleotide composition in the evolutionary divergence of Ascomycetes plant pathogens and paves the way to target specific codons and sequences to modulate host–pathogen interactions through genome editing and functional genomics tools.

## 1. Introduction

Regulation of genes at different levels, such as transcriptional, post-transcriptional, translational, and post-translational is critical for generating a functional product. Gene expression regulation is required for cellular differentiation, adaptation, development, and evolution [1,2]. The regulation of gene expression is mediated by protein molecules that bind to specific DNA sequences and act as an activator or repressor of gene expression, termed transcription factors (TF). Whole genome sequencing has led to the identification of ~80 transcription factor families in almost 200 fungal species [3]. Zinc finger proteins are one of the largest groups of transcription factors present in eukaryotes, with diverse secondary structures and functional characteristics [4]. These transcription factors have been categorised into three classes, namely Cys_2_His_2,_ Cys_4_ zinc finger, and C_6_ zinc finger proteins (Zn(II)_2_Cys_6_). C6 proteins comprise two zinc atoms bound to six cysteine residues and are termed and represented as zinc binuclear clusters Zn(II)_2_Cys_6_. These Zn(II)_2_Cys_6_ proteins are exclusively found in fungi and distributed in the ratio of 10:20:40 in chytrids-zygomycetes, basidiomycetes, and Ascomycetes, respectively [3]. Further, Zn(II)_2_Cys_6_ has been extensively studied in *S. cerevisiae*, with Gal4p as the signature protein [4], *Candida albicans* [1], *Tolypocladium guangdongense* [5], and *Aspergillus flavus* [6]. A wide range of functions has been attributed to Zn(II)_2_Cys_6_ proteins, from primary to secondary metabolisms, playing major roles in fungal development and imparting virulence to pathogenic fungi. It has been established that Zn(II)_2_Cys_6_ proteins are involved in carbon, nitrogen utilization, secondary metabolites biosynthesis, stress response [7], chromatin remodelling, melanin biosynthesis, sugar and amino acid metabolism, drug resistance [8], hyphal growth regulation, appressorium polarisation, etc. [9,10]. A mutant TPC1 (transcription factor for polarity control) *M. oryzae* strain showed delayed glycogen and lipid metabolism, along with appressorium-mediated plant infection [11]. In *Aspergillus oryzae*, transcription of *kojA* and *kojT* is regulated by the Zn(II)_2_Cys_6_ protein, KojR, which mediates the biosynthesis of kojic acid [12]. Similar orthologs were also identified in *A. flavus,* which, along with another gene cluster, regulates kojic acid biosynthesis [13]. In *A. nidulans*, almost 50 Zn(II)_2_Cys_6_ proteins have been identified with diverse roles such as involvement in ST biosynthesis (*AflR*), amylolytic gene expression (*AmyR*), conidial maturation (*VosA* gene), asexual and sexual development (*zcfA* gene, *FluG*) [14,15], etc. Mutation in *Ume6*, which is an *Zn(II)_2_Cys_6_*, leads to a defective hyphal extension in the host tissue, resulting in loss of virulence [16]. Two melanin biosynthetic genes, *SCD1* and *THR1*, are positively regulated by a Zn(II)_2_Cys_6_ protein named Cmr1p in *Collectotrichum lagenarium*, which infects cucumbers [17].

The genetic code consists of a set of 64 codons. Excluding three stop codons, the genetic code encodes for 20 amino acids. Except for methionine and tryptophan, most of the codons code for more than one amino acid. Codons that get translated into the same amino acid are referred to as synonymous codons. The usage of these synonymous codons is not uniform. The unequal or preferential use of synonymous codons leads to an inclination towards a specific set of codons called codon usage bias (CUB) [18]. CUB is widespread and involves variations among (1) amino acid codons, (2) genes of a genome, and (3) genomes of different species [19,20]. CUB is based on the theory of mutation-selection-genetic drift, which states that evolutionary forces generate some adaptive and non-adaptive mutations which do not affect the primary protein structure; however, CUB studies in model organisms suggested that CUB implants a marked influence on various transcriptional and translational processes [21]. The importance of CUB can be understood from the plethora of functions that influences, *viz.* mRNA transcription [22], mRNA stability [23], tRNA pairing [24], translational speed [25], correct protein folding [26], ensuring full protein biosynthesis [27], etc.

CUB has now been termed an important evolutionary parameter determining the expression of genes. The availability of novel and advanced sequencing techniques and genomic information resulted in an extensive study of CUB in both prokaryotes and eukaryotes. CUB is affected by factors such as hydrophobicity, gene length, replication, gene function, and secondary protein structure. Evolutionary factors contributing to CUB are mutational pressure and natural and translational selection [28]. It has been reported in some extremely AT-/GC-rich prokaryotic organisms, such as *Micrococcus luteus* [29], *Rickettsia prowazekii* [30], and *Borrelia burgdorferi* [31], that compositional bias solely governs the observed codon usage variations.

In contrast, there have also been instances in organisms, such as *Escherichia coli* [32], *Mycobacterium tuberculosis* [33], *Drosophila melanogaster* [34], and *Caenorhabditis elegans* [35], where translational selection pressure has been the major factor shaping codon usage signatures in highly expressed genes. Furthermore, codon usage patterns in eubacterial and archaeal genomes have also been reported to be a combinatorial consequence of mutational constraint and natural selection for translation [36]. Based on the large-scale data generated for various organisms, the researchers concluded that CUB is the result of balanced interaction of both natural selection and mutation pressure [20].

Through various studies, it has been established that CUB modulates heterologous gene expression, improves protein production, and regulates the cell cycle [37], cell proliferation and differentiation [38], and stress regulation [39]. Arella et al. [40] reported that CUB influences cellular fitness, which could further govern microbial organism ecology. Additionally, it was shown that CUB controls host–pathogen interactions by allowing the host and pathogens to adapt to their specific environments [41]. Codon optimization achieved through CUB favours pathogen colonisation in the hosts. Host colonization requires the secretion of certain diverse and complex proteins, evading host-imposed defence mechanisms and competing with other microbes [42]. The release of these secretory proteins is directly linked to translational efficiency, which is a crucial factor in synonymous codon usage patterns. Most of the studies conducted so far have focussed on the codon usage pattern at the whole genome level, especially in model systems and other species, *viz. Caenorhabditis*, *Escherichia coli*, *Drosophila*, *Arabidopsis*, *yeast*, *Giardia lamblia*, *Entamoeba histolytica*, *Ustilago*, *Borrelia burgdorferi*, *Taenia saginata*, *A. flavus*, *A. nidulans*, *Saccharomyces cerevisiae*, etc. [20,38,43,44,45,46]. However, there are hardly any studies comparing the CUB pattern of a gene family in plant–pathogenic fungi.

Ascomycetes pose highly deleterious effects on plants. Approximately 60% of top fungal pathogens belong to Ascomycetes [47] and are capable of causing 70–80% yield loss in crops [48]. The *M. oryzae* alone is responsible for causing 10–30% yield loss in rice [49]. The Food and Agricultural Organization reported that around 25% of the global food crops were contaminated by mycotoxins. Further, the harmful effects of Ascomycetes mycotoxins of plant pathogenic species from *Alternaria*, *Aspergillus*, *Fusarium*, and *Colletotrichum* extend to humans and animals [50]. These mycotoxins from Ascomycetes disrupt cellular functions and kill organisms, including humans, birds, and animals [51]. Additionally, the mycotoxin produced by *Fusarium* is one of the top five mycotoxins infecting humans [52]. The best-studied *Ochratoxin-A* is produced by several species of *Aspergillus*, and *Penicillium* is a common food-contaminating mycotoxin, especially in cereals, pulses, nuts, fruits, vegetables, and stored products [53]. Therefore, in the current investigation, *Zn(II)_2_Cys_6_* sequences unique to fungi were chosen to study the CUB patterns in nine Ascomycetes plant pathogenic fungi in relation to the model yeast system to decipher the association between CUB and evolutionary aspects shaping the Ascomycetes systems.

## 2. Materials and Methods

### 2.1. Mining of Zn(II)_2_Cys_6_ Sequences and Cluster Analysis

The whole-genome and proteome information of nine ascomycetous pathogenic fungi infecting various cereals, *viz. Alternaria alternata* (assembly: ASM415475v1), *Aspergillus flavus* (assembly: ASM245617v2), *Bipolaris maydis* (assembly: CocheC4_1), *Bipolaris oryzae* (assembly: *Cochliobolus miyabeanus* v1.0), *Colletotrichum graminicola* (assembly: *C. graminicola*_M1_001_V1), *Fusarium graminearum* (assembly: MDC_Fg13), *Gaeumannomyces tritici* (assembly: Gae_graminis_V2), *Pyricularia oryzae* (assembly: ASM434696v1), *Verticillium dahliae* (assembly: VdGwydir1A3), and model species *Saccharomyces cerevisiae* (assembly: R64-1-1) were downloaded from EnsemblFungi (https://fungi.ensembl.org/ (accessed on 3 May 2022)). The *Zn(II)_2_Cys_6_* sequences were identified employing HMM search with Pfam domain PF00172 (https://pfam.xfam.org/ (accessed on 3 May 2022)), and coding (CDS) sequences were retrieved from whole-genome CDS files of respective fungal species. The details of the number of CDS and codons studied are shown in Table 1. Further, all the retrieved CDS sequences (CDSs) were evaluated based on the following criteria to avoid short and partial sequences inducing bias [54]: (1) The minimum length of CDS was more than 300 bp; (2) The CDS should begin with a start codon ATG and end with any of the three termination codons, *viz*. TAA, TAG, and TGA; (3) CDS should be free from any internal termination codons. The cluster analysis based on *Zn(II)_2_Cys_6_* sequences was performed with ANACONDA v.2.0 software (https://bioinformatics.ua.pt/software/anaconda/ (accessed on 28 May 2022)) to visualize the species clustering behaviour based on *Zn(II)_2_Cys_6_* sequence composition.

### 2.2. Nucleotide Composition Analysis

The CDSs of ascomycetous pathogenic fungi of cereals under study and *Saccharomyces cerevisiae* were examined for nucleotide compositions. Nucleotide composition analysis was performed for each of the CDS sequences to quantify the frequencies of four standard nucleotides (A, T, G, and C), the occurrence of nucleotides at the third position of synonymous codons (A3, T3, G3, and C3), total GC content (GC%), and GC content at first (GC1), second (GC2), and third (GC3) positions of a codon. The percent GC content at the first and second position of codons (GC12) for each *Zn(II)_2_Cys_6_* CDSs was also calculated.

### 2.3. The Effective Number of Codons (ENC) and ENC Plot Analysis

The effective number of codons (ENC) considers the amino acid degeneracy level to calculate the total number of different codons used in a sequence. Therefore, ENC ranges from 20, with only one codon for each amino acid, to 61, with all the synonymous codons used with equal probability. Thus, ENC values are inversely proportional to codon usage bias. The ENC for *Zn(II)_2_Cys_6_* sequences were calculated as per Wright [48], as follows:

$$\mathrm{ENC}=2+\frac{9}{F_{2}}+\frac{1}{F_{3}}+\frac{5}{F_{4}}+\frac{3}{F_{6}}$$

where $F_{n}$ (n = 2, 3, 4, 6) is the mean of $F_{n}$ for n fold degeneracy of amino acids.

The ENC plots were generated by plotting the ENC values against the GC3 value of sequences. The ENC mainly determines whether a gene’s codon usage pattern is influenced by mutation and selection pressures. The position of ENC values of sequence on or around the standard GC3 curve suggests codon choice constraint owing to G + C mutation bias. If the ENC values are distributed considerably below the expected GC3s curve, this indicates the presence of selection effects on sequences [55].

### 2.4. Relative Synonymous Codon Usage Analysis

Relative synonymous codon usage (RSCU) is the ratio of the observed frequency of the codon to the expected usage frequency of all codons equally used within the given synonymous codon family of amino acids [56,57]. The RSCU of *Zn(II)_2_Cys_6_* CDSs from all the ten fungi species were calculated as per the following equation:

$$RSCU_{ij}=\frac{X_{ij}}{\frac{1}{n_{i}}\sum_{j=1}^{n_{i}}X_{ij}}$$

where *X_ij_* is the extent of *j*th codon for *i*th amino acid and *n*_i_ is the synonymous codons number for an *i*th amino acid.

### 2.5. Intrinsic Codon Deviation Index

Intrinsic codon deviation index (ICDI) provides a chi-square value independent estimate based on RSCU and degeneracy of amino acids in the sequence. ICDI is most helpful in estimating the codon bias in species where optimal codons are unknown [58,59]. The ICDI estimates are ranges from 0 with equal usage of all codons to 1 for one codon per amino acid. The ICDI estimates were calculated as per the following equation [38]:

$$ICDI=\underset{\alpha \in A}{\sum }F_{\alpha }S_{\alpha }$$

where $F_{\alpha }$ is a relative frequency of amino acid α and $S_{\alpha }=\frac{1}{k_{\alpha }\left(k_{\alpha }-1\right)}\underset{c\in C_{\alpha }}{\sum }{\left(r_{\alpha c}-1\right)}^{2}$. Here, $r_{\alpha c}$ is RSCU and $k_{\alpha }$ is the degeneracy of amino acid α.

### 2.6. Codon Adaptation Index

The codon adaptation index (CAI) quantifies the frequency or relative adaptiveness of a favoured codon being used amongst highly expressed genes. A codon’s relative adaptiveness (*w*) is calculated as the ratio of individual codon usage to that of the most abundant codon for the same amino acid [56]. Therefore, CAI of *Zn(II)_2_Cys_6_* CDSs are obtained through:

$$CAI=\overset{n}{\underset{k=1}{\prod }}w_{k}$$

where n is the number of codons and $w_{k}=\frac{RSCU_{i}}{RSCU_{max}}.$ Here, *RSCU*_max_ is the highest codon usage frequency for synonymous codons in a highly expressed reference gene, i.e., which represent the most abundant codon for an amino acid, and $RSCU_{i}$ refers to the relative occurrence of a unified codon of the first codon encoding the corresponding amino acids. CAI ranges from 0 to 1 and is a primary hint on translation efficiency [60].

### 2.7. Codon Bias Index (CBI)

The codon bias index (CBI) estimates the bias of the codon usage pattern of the coding sequence based on the degree of preferred codons. The CBI values range from 0 to 1. A CBI value of zero refers to a random choice of codons, whereas a CBI value of 1 indicates the sequence mostly uses preferred codons. The CBI of *Zn(II)_2_Cys_6_* CDSs were calculated using the following equation [61]:

$$CBI=\frac{N_{o}-N_{r}}{N_{t}-N_{r}}$$

where $N_{o}$ is the total occurrence of superior codons in the coding sequence, $N_{r}$ is the total of superior codons when all the synonymous codons are random, and $N_{t}$ refers to the frequency of amino acids corresponding to superior codons in the coding sequences.

### 2.8. Frequency of Optimal Codons (FoP)

The ratio of the number of optimal codons to the total number of codons (both optimal and non-optimal) provides the FoP index [32]. It is essential to understand that the FOP index is context- or species-dependent as its values depend on the genetic code of the particular species.

### 2.9. Synonymous Codon Usage Order (SCUO) Index

The synonymous codon usage order (SCUO) index quantifies the eccentricity from uniform distribution as a normalised difference between the maximum and observed entropy [62]. The average SCUO index for entire coding sequences was calculated using:

$$SCUO=\sum_{i=1}^{n_{i}}\left(\frac{\sum_{j=1}^{n_{i}}x_{ij}}{\sum_{i=1}^{18}\sum_{j=1}^{n_{i}}x_{ij}}\right)SCUO_{i}$$

where *j* is the codon *i*th amino acid and $SCUO_{i}=\frac{H_{i}^{max}-H_{i}}{H_{i}^{max}}$. Here, $SCUO_{i}$ is the SCUO for *i*th amino acid in each sequence and $H_{i}$ and $H_{i}^{max}$ are the entropy and maximum for an *i*th amino acid in a sequence.

### 2.10. Codon Usage Similarity Index

The codon usage similarity index (COUSIN) compares the codon usage preferences of a query sequence with the reference and normalises the output over the assumption of the null hypothesis of random codon usage. The COUSIN could be computed as COUSIN_18_ or COUSIN_59_. The COUSIN_18_ allows the equal contribution of each of the 18 families of synonymous codons to the global index. In comparison, COUSIN_59_ allows the proportional contribution of each family to the frequency of the corresponding amino in the query sequences. The COUSIN scores of *Zn(II)_2_Cys_6_* CDSs of all the fungi species were computed using COUSIN software [63]; (https://cousin.ird.fr (accessed on 15 May 2022)).

### 2.11. GRAVY and AROMA

The biochemical properties of the final hypothetical translated products, viz., hydropathicity and aromaticity, are associated with codon bias of coding sequences. The general average hydropathicity or the grand average of hydropathicity (GRAVY) score was employed to estimate the hydropathy of sequence. GRAVY is calculated as the arithmetic mean of the sum of the hydropathic indices of each amino acid in a hypothetical translated coding sequence product. The positive and negative GRAVY scores the hydrophobic and hydrophilic nature of the protein [64]. The aromaticity score provides the frequency of aromatic amino acids (Phe, Tyr, and Trp) in the hypothetical translated coding sequence product [65].

### 2.12. PR2 and Neutrality Plots

PR2-bias plots were generated based on the principle of parity rule 2. The parity rule 2 (PR2) states that under the absence of selection and mutational pressure, the nucleotide bases follow the A = T and G = C (where A + T + G + C = 1) rule [66]. The A3/(A3 + T3) and G3/(G3 + C3) values of every *Zn(II)_2_Cys_6_* CDS sequence were calculated and used as the ordinate and abscissa to visualise the association between purine (A and G) and pyrimidine (T and C) at the third codon position in the form of a PR2 bias plot. When A = T and G = C (PR2), the centre of the plot where both coordinates are 0.5 harbours the data points. Therefore, any deviation from the centre of the PR2 plot allows estimating the chain bias affected by the mutation, selection, or both. The significant deviation from the parity rules at the third codon position of four-codon amino acids mostly results from selective biases rather than mutational biases during evolution. In other words, if the data points are evenly distributed across the plan view, that is, if the frequency of A + T is equal to that of G + C at the third position of the codon, then the codon usage preference mainly results from mutation [66,67].

The neutrality plots for *Zn(II)_2_Cys_6_* CDSs were generated by plotting the average GC1 and GC2 (GC12) values against GC3. The neutrality plots depict the effect of mutation-selection equilibrium in shaping the codon usage bias of sequences [68]. In neutrality plots, regression with a slope of 0 suggests the absence of directional mutation pressure or complete selective constraints. On the other hand, a slope of 1 indicates the same mutation module between GC12 and GC3 and that complete neutrality was the main element in the evolutionary process [69].

### 2.13. Translational Selection Index (P2)

The translation selection index (P2) provides the efficiency of codon–anticodon interactions and indicates translation efficiency if the information on preferred codon sets is unavailable. The P2 values were calculated with the following formula: P2 = (WWC + SSU)/(WWC + SSY), where W = A or U, S = C or G, and Y = C or U [70]. A P2 value of more than 0.50 (P2 > 0.50) indicates the preference for translational selection in the given coding sequence.

### 2.14. Correlation and Principal Component Analysis

The association of nucleotide compositions with various codon bias parameters and RSCU of *Zn(II)_2_Cys_6_* CDSs were investigated through correlation analysis employing SAS 9.2. The principal component analysis was employed to realise the correlations between sequences and codons. After removing the terminal and start codons, viz., UAA, UAG, UGA, UGG, and AUG from every *Zn(II)_2_Cys_6_* CDS, the data was represented as a 59-dimensional vector, where each dimension corresponded to each sense codon’s RSCU [18,71]. The PCA plots were generated with Origin 8.5 (OriginLab, Northampton, USA) software.

## 3. Results

### 3.1. Nucleotide Composition Analysis

Detailed knowledge about the nucleotide composition of a coding sequence provides a basis for understanding the codon distribution across genes or species and its association with gene activity. Individual nucleotide composition, frequency of nucleotides at the third position, and overall composition was studied for all the ten target species. The frequency of nucleotide C (cytosine) was highest in all species, followed by A (adenine), G (guanine), then T (thymine). Out of all the four nucleotides, cytosine was the most available nucleotide, with an average value of 28.62 ± 4.04, followed by guanine (25.16 ± 2.91), adenine (24.47 ± 3.73), and thiamine (21.75 ± 3.22). An overall analysis of GC and AT composition showed the predominance of GC-richness in *Zn(II)_2_Cys_6_* coding sequences. However, *C. graminicola*, *G. tritici*, *P. oryzae*, and *V. dahliae* showed a higher percentage of GC than other species. Compared with the Ascomycetes group of fungi, *S. cerevisiae* showed high AT-richness (61.56%), and only 38.44% was contributed by GC percentage (Table 2). The nucleotide type present at the third position of the codon has been known to be a key determinant of the amino acid; therefore, nucleotide composition at the third position was also critically investigated. Interestingly, at the third position of the codon, cytosine was the most preferred nucleotide, i.e., among GC3 and AT3; cytosine was the most frequently present nucleotide, followed by T, G, and A. *G. tritici* had the highest GC3%, followed by *V. dahliae*, *C. graminicola*, and *P. oryzae*. The nucleotide composition of *Zn(II)_2_Cys_6_* coding sequences of all the target species is given in the Supplementary Information (Tables S1–S10).

### 3.2. Relationship between Fungal Species via Clustering Analysis with Zn(II)_2_Cys_6_ Coding Sequence Parameters

**Figure 1:**
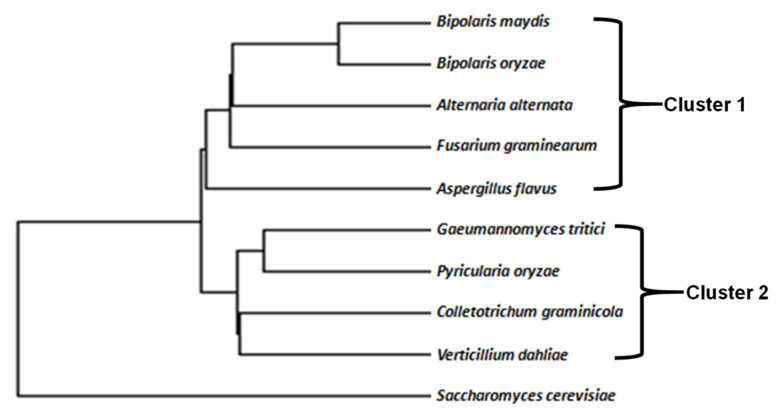
Cluster analysis of target fungal species based on *zinc binuclear cluster* coding sequences, nucleotide composition, and codon context parameters by ANACONDA 2.0.

The clustering analysis divided the target fungal species into two major groups, with *S. cerevisiae* as an outlier. The first branch contained nine plant pathogenic fungal species belonging to Ascomycetes. The major branch with Ascomycetes was bifurcated into two clusters. The first cluster contained five species, *viz. B. maydis*, *B. oryzae*, *A. alternata*, *F. graminearum*, and *A. flavus*, while the other contained *G. tritici*, *P. oryzae*, *C. graminicola*, and *V. dahliae* (cluster 2). Separate positioning of *S. cerevisiae* from other fungal species may be attributed to AT abundance in the *Zn(II)_2_Cys_6_* sequences, in contrast to GC-richness in fungal species belonging to the Ascomycetes group (Figure 1). Further clustering of fungi was very well-correlated with the CUB indices, where both groups of fungi showed similarities within their groups in terms of values and results, as shown in later subsections. The number of over-represented GC-rich codons was greater in a group comprising *G. tritici*, whereas there were more AT-rich codons in a group comprising *B. maydis*.

### 3.3. Relative Synonymous Codon Usage Analysis

**Figure 2:**
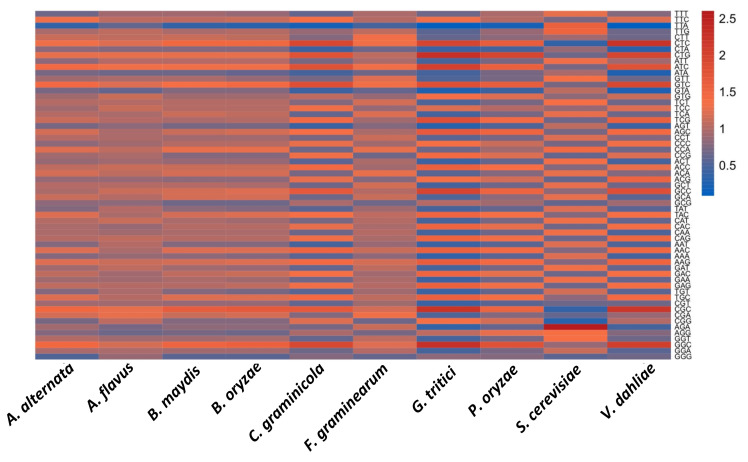
The heat map showing the relative synonymous codon usage (RSCU) in zinc binuclear clusters in various Ascomycetes plant pathogenic fungal species and model yeast systems.

To get an insight into codon usage variation, RSCU analysis was conducted, and subsequently, data were classified into different groups based on RSCU values: (1) RSCU > 1.6 were considered to be overrepresented codons or those with a strong preference; (2) RSCU between 1–1.6 were considered high usage frequency of the codon; (3) RSCU between 0.6–1 represented less frequently used codons; (4) RSCU < 0.6 were considered underrepresented. In all the 10 species studied, the presence of A/T rich and G/C rich codons was close to 50%, i.e., either 29 or 30 out of 59 codons. Most high-frequency codons were GC-rich except in *S. cerevisiae*, where the preference was more toward A or T codons. Similarly, overrepresented codons were most GC-rich except in *S. cerevisiae*, where all the six strong preferred codons were A/T rich (TTA, AGT, CAA, AAA, TGT, and AGA). *G. tritici* showed the maximum number of codons with RSCU values below 0.6 (23 A/T rich codons) and above 1.6 (10 G/C rich codons), respectively. Out of the nine Ascomycetes species, the four species showing maximum overrepresented codons belonged to cluster 2, which is already known to be composed of high GC-richness (Table 3). The frequency of underrepresented codons was more for A/U rather than G/C ended codons, which happened to be 58 and 7, respectively, in a total of all species. Further, the detailed RSCU study revealed that among 59 codons, 10 codons (7 GC-rich codons: CTC, CTG, GTC, GAG, CGC, TGC, and GGC; 3 AT-rich codons: TTC, ATC, and AAG) were either overrepresented or of high usage and were present in all the fungal species except for *S. cerevisiae,* and 5 (4 GC-rich codons: AGC, GAC, GCC, and ACC; 1 AT-rich codon: TAC) were present in eight of the ten fungal species (Table S11). The complete list of RSCU values for each codon in each species is shown in Table S11 and Figure 2. Sharing the same set of GC-rich codons (CTG, GTC, GAG, and CGC) by all the fungal species belonging to different orders of Ascomycetes highlights the importance of these codons in determining codon usage patterns of *Zn(II)_2_Cys_6_* sequences. In addition, it reveals that *Zn(II)_2_Cys_6_* genes have greater preference for G/C-ended codons in comparison with A/T-ended codons.

### 3.4. ENC and ENC Plot

ENC is a parameter used to determine the degree of CUB in a given sequence. ENC values below 35 signifies high codon preference, and above 50 reveal random codon usage [72,73]. The average ENC values of *Zn(II)_2_Cys_6_* CDSs of the target fungal species ranged from 44.33–58.65, indicating slightly random CUB to no strong codon bias. Further, none of the *Zn(II)_2_Cys_6_* sequences among all the species, except for *C. graminicola* (3), *G. tritici* (14), and *V. dahliae* (7), had ENC values below 35, indicating the predominance of random codon usage patterns (Tables S1–S10). An inverse association was reported between the codon preference ENC value and gene expression, i.e., a low ENC value means a higher preference for codon bias and higher gene expression, and *vice versa* [72,73]. Correlation coefficient analysis showed a negative correlation between ENC and GC3, with *C. graminicola*, *G. tritici*, *V. dahliae*, and *P. oryzae* (cluster 2) being the most strongly negatively correlated compared with other fungal species.

As the GC content of the gene is an important determinant of ENC, an ENC plot was developed to understand the effect of GC3 on codon bias. If mutation was the sole factor responsible for codon bias, then genes were distributed either on the standard curve or above it, which also signified that genes were showing no bias, whereas if codon bias was affected by selection, then genes lay sufficiently below the standard curve [55,73]. Some of the *Zn(II)_2_Cys_6_* sequences were present on or above the standard curve, which implied that the compositional constraint was one of the essential factors in dictating codon usage, as was evident from the ENC plot of species in cluster 2, *viz. C. graminicola*, *G. tritici*, *V. dahliae*, and *P. oryzae* species.

**Figure 3:**
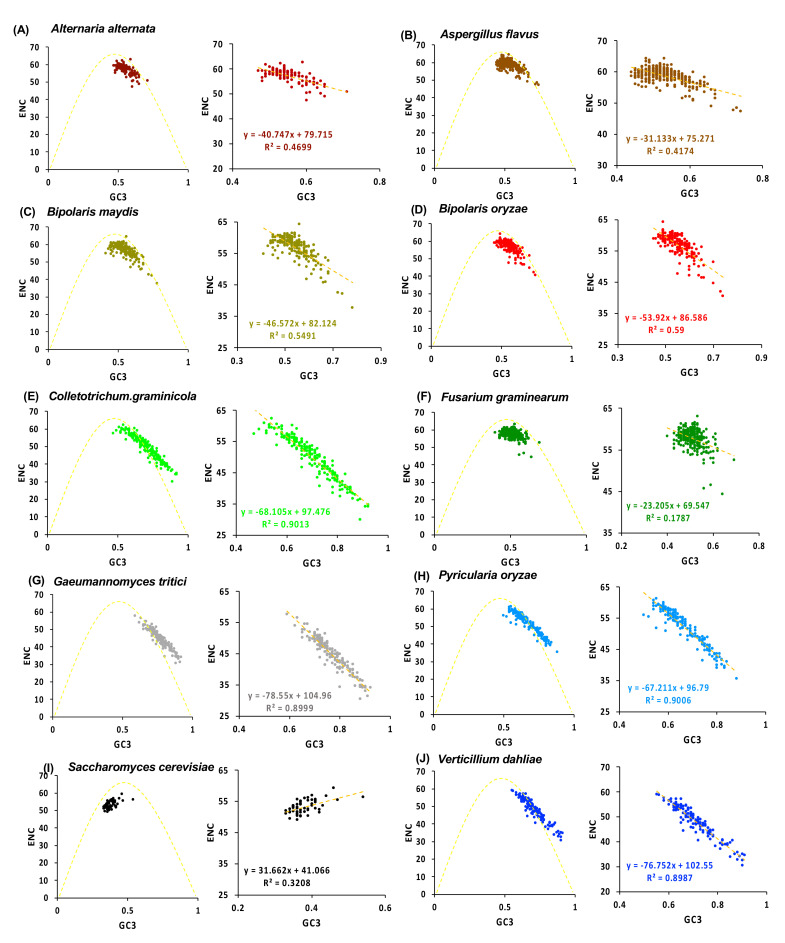
The effective number of codons (ENC)-plot showing a relationship between ENC and GC3s in coding sequences of *zinc binuclear cluster* family in the target fungal species.

On the contrary, in *A. alternata*, *A. flavus*, *B. maydis*, *B. oryzae*, *F. graminearum* (cluster 1), and *S. cerevisiae*, the genes clustered slightly below the standard curve, suggesting not only compositional constraint, but natural selection and other factors played a minor role in determining codon usage patterns (Figure 3). The result was in concordance with studies conducted for the whole genome of the genus *Ustilago*, *Epichloe festucae*, *Meloidogyne incognita*, and *A. alternata* [46,74,75]. The presence of a GC3 distribution in the range of 0.4–0.9, with *S. cerevisiae* as an exception, further strengthened the idea of the effect of mutation pressure on codon usage. These current findings were supported by the results of Kawabe and Miyashita [76], in which the GC3 distribution was a deciding factor between directional selection and mutational pressure.

### 3.5. Intrinsic Codon Deviation Index (ICDI)

**Figure 4:**
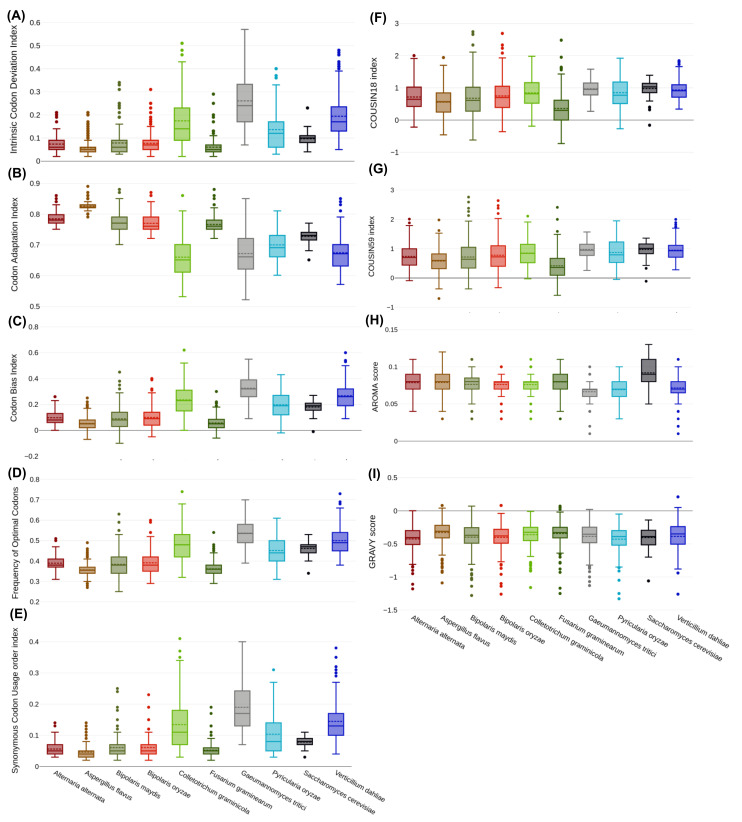
The box and whisker plots depict the descriptive statistics of various codon bias parameters. Each box plot with distinct colour indicates one species. Brick red, mustard yellow, olive, red, light green, dark green, grey, sky blue, black, and purple colour represent *A. alternata*, *A. flavus*, *B. maydis*, *B. oryzae*, *C. graminicola*, *F. graminearum*, *G. tritici*, *P. oryzae*, *S. cerevisiae*, and *V. dahliae*, respectively. The diagrams include: (**A**) ICDI, (**B**) CBI, (**C**) CAI, (**D**) FoP, (**E**) SCUO, (**F**) COUSIN18, (**G**) COUSIN59, (**H**) AROMA score, and (**I**) GRAVY score.

ICDI is another tool to measure codon usage bias with values of 0 to 1. The genes possessing an ICDI value between 0.3–0.5 are moderately expressed, which means that an ICDI value below 0.3 signifies lower gene expression, which is related to low codon bias, whereas an ICDI value above 0.5 has higher codon bias, hence high gene expression. In the present study, the overall mean ICDI was 0.06–0.26 ± 0.069, which suggested that *Zn(II)_2_Cys_6_* coding sequences have a low codon bias (Figure 4A). Despite all the species showing low biasness, if an attempt to compare both the clusters was made, higher values were seen for cluster 2 than 1. This could be linked to ENC results of high and low codon biasness, whereas in ENC analysis, cluster 2 also showed relatively more biasness than cluster 1. The results for *S. cerevisiae* were intermediate between both clusters.

### 3.6. Codon Adaptation Index (CAI)

CAI is a measure of adaptation of synonymous codon usage of a gene with respect to a reference set of the gene; in other words, it assesses the merits of preferred codons in highly expressed genes [56]. The range set for CAI is 0–1, where a value of 1 corresponds to a gene that utilises a specific set of codons, thereby supporting high codon usage bias. CAI values for *Zn(II)_2_Cys_6_* sequences varied from 0.651–0.828 ± 0.059, with *V. dahliae* showing minimum CAI and *A. flavus* showing maximum CAI (Figure 4B). Based on the CAI value, it can be postulated that *Zn(II)_2_Cys_6_* sequences are highly expressed, as it is directly associated with gene expressivity, gene expression levels, adaptation, and codon usage bias [73,77,78].

It has been suggested in many studies that transcription factors belong to the category of essential genes and are also highly expressed. The function of these genes is closely related to optimal codon composition, as it can cut down energy costs and make the gene biologically significant [44]. As stated previously, the zinc binuclear protein family belong to the transcription factor category; thus, it can be inferred that zinc binuclear proteins are highly expressed genes that are well-correlated with high CAI values [4]. A high negative correlation between CAI and ENC further validates our idea of increased gene expression. Simultaneously, a significant positive correlation was also observed with GC and GC3 content (for GC r = 0.55–0.88 and GC3 r = 0.59–0.95), which indicates that codons in *Zn(II)_2_Cys_6_* sequences are GC-rich. This can be correlated with RSCU values where codons with RSCU > 1 were mainly GC-rich, implying that the gene preferred optimal codons ending with cytosine and guanine over uracil and adenine. An exception to the current observation was *S. cerevisiae*, which favoured AT-rich codons rather than GC, and showed a positive correlation with AT and AT3. Perseverance of GC-rich codons facilitates pathogen invasion in the host system by promoting gene expression, and this richness of G and C is common in fungal genomes [46,79].

### 3.7. Codon Bias Index (CBI), FoP, SCUO, and COUSIN

Different fungal species had different CBI values; however, they held uniformity in terms of random usage of preferred and non-preferred codons. *A. flavus* had the least CBI value of 0.053, whereas the maximum was for *G. tritici* (0.325), i.e., a member of cluster 2 (Figure 4C). The results suggest low usage of highly expressed codons [54]. FoP (frequency of optimal codons) is also a measurement of usage of preferred or non-preferred codons. A value near 1 is indicative of utilization of preferred codons, whereas a value closer to 0 signifies the rare appearance of optimal codons. In our case, FoP ranged from 0.356–0.536, which could be interpreted as a lower inclination toward optimal codons. However, the FoP value for cluster 2 was greater than for cluster 1 (Figure 4D). SCUO was calculated to determine codon biasness, and it was found that values were close to 0, indicating less codon biasness. The values were in the range of 0.046–0.189, with an average of 0.092 ± 0.048 (Figure 4E). The COUSIN index, being another determinant of biasness, revealed that there was a weak to moderate codon biasness, as values corresponding to 0 show equal usage of synonymous codons, 1 shows high codon usage preference, and between 0–1 shows weaker biasness. For the present analysis, the value of the COUSIN index was between 0–1, i.e., 0.367–0.984 (Figure 4F,G). These CUB indices showed that, in comparison to cluster 1, cluster 2 had a higher degree of codon biasness.

### 3.8. GRAVY and AROMA

Aromaticity (AROMO) values of target fungal species showed an approximately equal proportion of aromatic amino acids with a range of 0.066 to 0.091. *S. cerevisiae* showed the highest aromaticity with a value of 0.091 (Figure 4H). GRAVY scores determine the hydropathy of a protein. Positive and negative values represent the hydrophobic and hydrophilic nature, respectively. For all the fungal species, the mean values of GRAVY were almost similar and in the range of −0.430 to −0.327 (Figure 4I). The mean negative value indicated that the *Zn(II)_2_Cys_6_* sequences were predominated by codons which coded hydrophilic amino acids.

### 3.9. PR2 Plot Analysis

**Figure 5:**
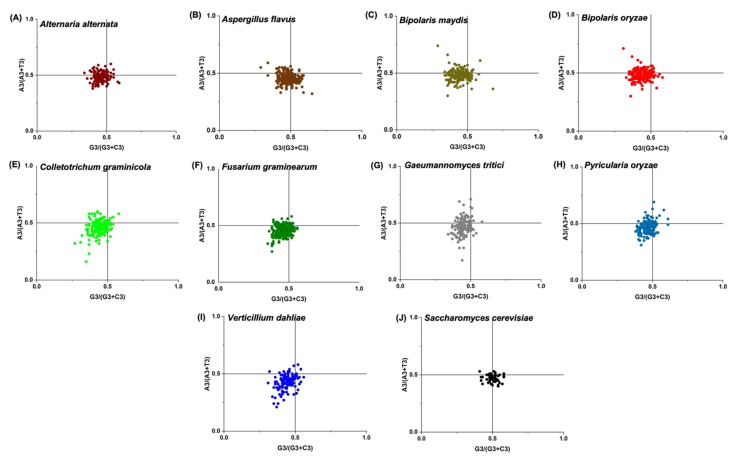
Parity rule (PR2)-bias plot analysis of *zinc binuclear cluster* coding sequences in Ascomycetes plant pathogenic fungi and model yeast species.

Mutational force and natural selection are the two important factors shaping the current CUB of coding sequences. The presence of mutational force and natural selection on CUB of sequences was ascertained by the PR2 bias plot analyses. The PR2 bias plot analysis of *Zn(II)_2_Cys_6_* sequences showed that most data points in plant pathogenic Ascomycetes fungi were plotted in the lower left quadrant of the parity plot, showing that T and C were the nucleotides of choice in the target coding sequences. As these phytopathogens were GC-biased and the PR2 plot showed biasness towards T and C, a general biasness towards C-ending codons was observed (Figure 5A−I).

Contrary to other fungi, in *S. cerevisiae,* the distribution was in the left and right lower quadrants in the PR2 plot, which showed selectivity towards T-ending codons (Figure 5J). These results could very well be justified by the nucleotide composition analysis, where cytosine was the predominant nucleotide in the *Zn(II)_2_Cys_6_* sequences of the Ascomycetes group and T for *S. cerevisiae*. As the codons do not occupy the centre position in the plot but are deviated from the centre, it is evident that the observed CUB in *Zn(II)_2_Cys_6_* sequences is not only the function of mutation pressure but also selection pressure. The same was also evident in the case of *A. alternata* [68]. Further, the dual effect of natural selection and mutation pressure on the dispersal of codons from the centre of the PR2 plot was confirmed in the *TP3* gene family [38], *Zingiber officinale*, and its associated fungal pathogens [79].

### 3.10. Neutrality Plots Analysis

**Figure 6:**
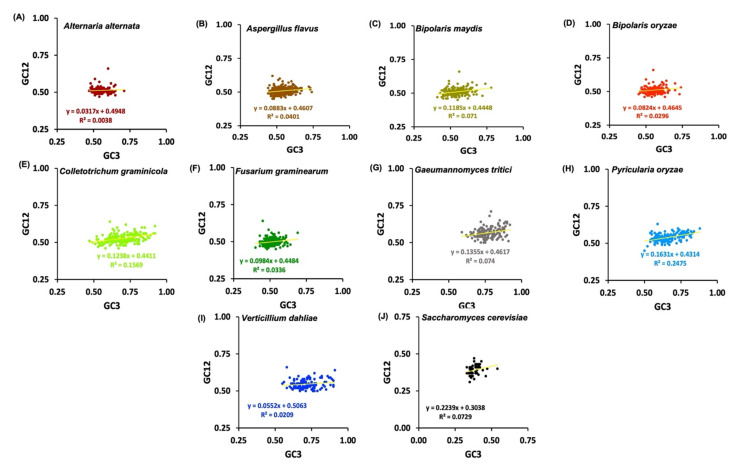
Neutrality plots plotted with GC12 vs. GC3 content of *zinc binuclear cluster* sequences in different species of Ascomycetes family and yeast.

The neutrality plot elucidated the relationship between GC12 and GC3 to determine the influence of mutational pressure and natural selection on CUB usage (Figure 6). The neutrality plot in our study for all the Ascomycetes species showed that the *Zn(II)_2_Cys_6_* genes exhibited a wide range of GC3 values, ranging from 48–92%, whereas for *S. cerevisiae,* this range started from 33%, which was an indication of the effect of dual forces. The slope of the regression for all the fungi was less than 1, i.e., 0.031 (*A. alternata*), 0.088 (*A. flavus*), 0.118 (*B. maydis*), 0.082 (*B. oryzae*), 0.123 (*C. graminicola*), 0.098 (*F. graminearum*), 0.135 (*G. tritici*), 0.163 (*P. oryzae*), 0.223 (*S. cerevisiae*), and 0.055 (*V. dahliae*) (Figure 5), which meant that the effect of mutation pressure was 3.1, 8.8, 11.8, 8.2, 12.3, 9.8, 13.5, 16.3, 22.3, and 5.5%, respectively. These values indicate that codon bias was affected less by mutational pressure and more by natural selection. Further, there was no significant correlation between GC12 and GC3, which further confirmed the supremacy of natural selection over mutational pressures [36,68].

### 3.11. Translational Selection Index (P2)

The interaction efficiency of codon–anticodon was screened by P2 analysis, where a value above 0.5 indicated the pronounced effect of translational selection during codon usage. Data generated revealed that for *C. graminicola*, *G. tritici*, *P. oryzae*, *V. dahliae* (cluster 2), and *S. cerevisiae* (Table 4), the values were less than 0.5, which meant that mutational pressure showed more influence on CUB of *Zn(II)_2_Cys_6_* sequences compared with other species. This was consistent with the high GC and GC3 content, except for *S. cerevisiae*, which was AT-rich. In *A. alternata* and *F. graminearum* (cluster 1) (Table 4), this value was greater than 0.5, which gave a clear indication of the higher influence of translational selection. For *A. flavus*, *B. maydis*, and *B. oryzae* (cluster 1) (Table 4), the P2 was equal to 0.5; however, this was the mean of P2 values of the number of CDS. When P2 values for each CDS of these species were analysed, it was found that a higher number of CDS had P2 > 0.5, which indicated that these species were more inclined toward translational selection (Table S12).

### 3.12. Principal Component Analysis

**Figure 7:**
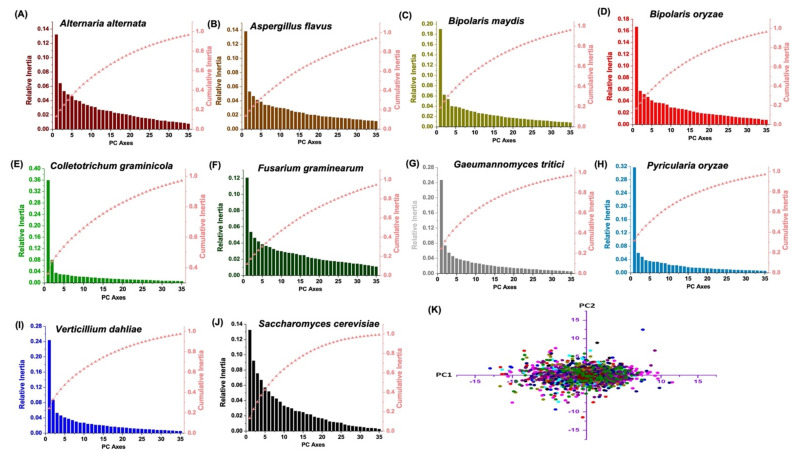
The principal component analyses of RSCU values of zinc binuclear cluster family coding sequences. (**A**–**J**) Inertia value of the axes obtained from the PCA analysis of the RSCU values for zinc binuclear cluster family coding sequences from the ten target species under study: (**K**) Plotting the principal axes values explaining maximum variances from all the ten species.

Principal component analysis was conducted to determine the trends in codon usage for *Zn(II)_2_Cys_6_* sequences. It was visualised that axis 1 and axis 2 were the major contributors to variance, followed by axis 3 and 4; the remaining axes hold less responsibility for codon usage variation. Axis 1 accounted for the maximum variation in the range of 12.06–36.01%. The contribution bestowed by both axes in each fungal species is listed in Table S13, which shows that, compared with *F. graminearum*, *A. alternata*, and *A. flavus* (cluster 1), axis 1 had a more pronounced effect in *G. tritici*, *C. graminicola*, and *P. oryzae* (cluster 2). Each of the coloured circles represent an individual *Zn(II)_2_Cys_6_* gene, with each colour being representative of a fungal species. The circles lay across the four quadrants, mainly concentrated near the axis; also, there was an instance of overlapping within the fungal species (Figure 7). Despite differences in sequences, all the fungal species shared similar codon usage patterns, to an extent. The incidence of some circles scattered away from the axis may be marked as the effect of other evolutionary forces, such as natural selection.

### 3.13. Correlation Analysis of CUB Indices

A scrupulous study was conducted to ascertain the relationship between different CUB indices, which would help to understand the pattern of codon usage and the factors influencing it. It has already been established that CAI and ENC share a negative relationship. CAI strongly correlated with overall GC content, individual GC1, GC2, GC12, GC3 components, and FoP. The maximum r value for GC3/CAI correlation was r = 0.97 *** for *G. tritici,* and Fop/CAI correlation was r = 0.97 *** for *C. graminicola*. The relation between FoP and GC3 was also positive. The CAI/FoP marked a positive correlation with GC-rich indices; however, they showed a strong negative correlation with AT and AT3 (Table S13–S22). All the fungal species showed a similar correlation pattern except for *S. cerevisiae*, which responded in an opposite manner to Ascomycetes fungal counterparts, i.e., CAI and FoP were positively correlated with AT and AT3 and negatively correlated with GC, GC1, GC2, and GC3. For the ENC parameter, it showed a positive correlation to AT and AT3, whereas is had a negative correlation with CAI, FoP, GC, GC1, GC2, and GC3 for all nine Ascomycetes species with varying r values (Tables S13–S22). For ENC, the results for *S. cerevisiae* were contrary to what was found for the other fungal species. Analysis of its relationship with other parameters showed a strong negative correlation with ENC, where it had a positive relation with CAI, Fop, ICDI, and GC3 indices. The positive relation between SCUO and GC3 was more pronounced in the fungal species of cluster 2 than cluster 1, signifying the role of mutational pressure on CUB in these species. The COUSIN indices exhibited a strong positive relation with CAI, SCOU, and GC3 and a negative relation with ENC and axis 1, implying that compositional constraints played a role in CUB determination.

The GRAVY and AROMA scores were also correlated with other CUB Indices. The correlation of GRAVY and AROMA scores with other CUB indices was variable among the ten species. Gene length showed a significant negative correlation with GRAVY in *A. alternata*, *A. flavus*, *C. graminicola*, and *S. cerevisiae* and was significantly positively correlated with AROMA for *A. alternata*, *A. flavus*, *B. maydis*, *G. tritici*, *P. oryzae*, and *V. dahliae*; however, the association in other species were nonsignificant (Tables S14–S23). Axis 1 exhibited a considerable positive correlation with GRAVY for cluster 1, except *A. flavus,* and a negative correlation existed in *P. oryzae* and *V. dahliae*. On the other hand, no significant correlation was observed between axis 1 with AROMA and between axis 2 with either AROMA or GRAVY scores. AROMA had no significant relation to ENC and CAI. At the same time, hydrophobicity was positively correlated to ENC for cluster 1, except *A. flavus*, and negatively correlated with *P. oryzae* and *V. dahliae*, and *vice versa* for CAI. The results indicated that hydrophobic proteins had weaker codon bias for cluster 1 and stronger codon bias for species of cluster 2. ENC values showed strong positive correlation with axis 1 (r = 0.67–0.97, *p* > 0.01) and equally strong negative correlation coefficients with CAI (r = −0.63 to −0.97, *p* > 0.01). Thus, it can be inferred that ENC may be one of the major factors in determining codon bias. The influence of CDS length on axis 1, CAI, and ENC could not be adequately determined as the correlation was significant for some species and nonsignificant for other species. However, based on the available information, gene length was positively correlated with CAI and negatively correlated with axis 1 genes with a longer length and higher expression level occupying the right side of the first axis. This observation was found for *A. alternata*, *C. graminicola*, *F. graminearum*, and *S. cerevisiae*, showing significant values. Conclusively significant correlations between AROMA, GRAVY, ENC, CAI, and axis 1 suggest an influential role of translational selection, especially in cluster 1 species, which was consistent with translational selection index results.

## 4. Discussion

The strong relation of CUB indices with GC3 was evident through CAI, ICDI, and FoP analyses, suggesting an important role of compositional constraint in determining codon biasness. Codon usage is known to be shaped by nucleotide composition [80]. In our study, we found that cytosine was most prominent among all the nucleotides (overall, as well as at the 3rd position). All of the nine Ascomycetes plant pathogenic fungi exhibited a high level of GC% and GC3%, despite having varying levels of GC-richness, i.e., cluster 2 was more GC-rich than cluster 1 (Table 2). This implied that depending on the recombination rate, GC heterogeneity and GC bias did influence CUB [81,82]. Furthermore, through RSCU analysis, we found that the most preferred and overrepresented codons mainly were GC-rich with cluster 2 (Table 3). Sharing of ten highly preferred codons by all the nine Ascomycetes, out of which seven were GC-rich codons, CTC, CTG, GTC, GAG, CGC, TGC, and GGC, and three AT-rich codons, TTC, ATC, and AAG, was direct evidence of the conservation of these codons during the course of evolution and the importance of these codons for *Zn(II)_2_Cys_6_* expression (Table S11). *S. cerevisiae* was found to be AT-rich, and this variation was responsible for keeping it out of the clusters of Ascomycetes. However, results from the neutrality plot, ENC plot, and PR2 showed different aspects of the story. The neutrality plot can be referred to as a tool to establish the influence of mutation pressure over natural selection. The ratio of mutational pressure to natural selection turned out to be 0.03 (*A. alternata*), 0.10 (*A. flavus*), 0.13 (*B. maydis*), 0.09 (*B. oryzae*), 0.14 (*C. graminicola*), 0.10 (*F. graminearum*), 0.15 (*G. tritici*), 0.19 (*P. oryzae*), 0.28 (*S. cerevisiae*), and 0.05 (*V. dahliae*) (Figure 6). The low ratios indicated that the codon usage pattern was driven more by natural selection and less by mutational forces. Several organisms have been studied where CUB is more often a function of natural selection, such as in *Calypogeiaceae*, *Marchantiophyta*, and others [83,84]. Drifting codons from the centre and not concentrating at the centre can be further associated with the involvement of forces other than mutational pressure, as shown in PR2 plots (Figure 5). ENC plots clarified the involvement of evolutionary forces responsible for biasness where the occurrence of genes below the standard curve indicated the role of natural selection along with compositional constraint (cluster 1). In contrast, being on or above the curve indicated an influential role of mutational pressure (cluster 2). P2 analysis data also showed a mutual role, and similar with the ENC plot, both fungal clusters exhibited different levels of forces acting on them. Cluster 2 were majorly P2 < 0.5 and cluster 1 were mostly P2 > 0.5, indicative of the idea that CUB may vary for the same gene family across the genomes. Overall, it can be inferred that codon biasness results from dual forces with a major impact posed by natural selection. Our results are in accordance with various other studies on eukaryotes and prokaryotes [47,75,85]. Additionally, it has been suggested that CUB results from a mutual partnership between selection and mutation, which is balanced by various unknown forces at different levels of organisation [19,20].

A strong association of GC3 with SCUO confirmed the variation of codon usage orders among the fungal species and that GC3 was one of the key determinants of codon bias in these *Zn(II)_2_Cys_6_* proteins. As the GC biasness increases, the CUB also increases. The positive correlation between GC3, overall GC, and SCUO has also been reported in previous studies [86].

Critical analysis of all the CUB indices in the ten species highlighted the association between evolution and codon usage patterns. Clustering analysis resulted in the generation of two branches, with *S. cerevisiae* as an outlier. The first or major branch was divided into two clusters. Interestingly the results of the CUB analysis could also be divided into two parts which coincided well with the clustering pattern of species. For instance, GC-richness, CAI, FoP, and SCUO were greater in all four species of cluster 2, whereas incidences of translational selection with a higher P2 index were greater in all five species of cluster 1. The results can be justified by the argument that CUB is the result of collective actions of mutation, natural selection, and genetic drift, which shape the evolution of genomes [87]. CUB reflects the origin, mutation, and evolution of genes and can be used to determine the evolutionary pattern among genes, species, organisms, etc., as closely related organisms are expected to have similar CUB patterns [81,88]. The various species of fungi used in the present study are the causative agents of devastating plant diseases, such as leaf spot (*A. alternata*), southern corn leaf blight and stalk rot (*B. maydis*), blast (*P. oryzae*), etc. The Zn(II)_2_Cys_6_ protein is one of the potential causative factors for inducing infection in a plant. It is an exciting target to study in terms of its codon usage in these fungi. Badet et al. [42] reported that optimal codons channel the adaptation and colonisation of parasites to their respective hosts. Less information is available on plant–fungal interactions; however, extensive work has been conducted on viruses and parasites based on the importance of codon optimisation for *Zn(II)_2_Cys_6_*. *Myco-reovirus* isolated from *Cryphonectria parasitica* (chestnut blight fungus), along with other *myco-reovirus*, showed evidence of codon biasness for XYG+XYC and established that CUB in reovirus, and their respective hosts, would have been adapted during evolution [89]. Selection of optimal codons to adapt themselves to their host environments is an integral part of viral evolution, as was evident from various studies conducted on *reoviruses*, bacteriophages, mammalian viruses, plant viruses, etc. [89,90,91,92]. In nature, the sharing of synonymous CUB patterns by the host plants and their respective pathogens could be an outcome of common mutational bias or natural selection driven by evolutionary forces. Similarity in CUB patterns was observed between dicot plants and infecting viruses [93]. The same kind of codon usage adaptation of pathogens toward plants was also perceived by fungal systems. For instance, interaction of *Crocus sativus* with *Aspergillus fumigatus* and *Fusarium oxysporum*, and *Z. officinale* with *A. flavus*, *A. niger*, and *F. oxysporum* showed similar CUB indices [79,94]. ENC and CAI are the critical determinates of gene expression level, indicating the important role of CUB in deciding expression of genes. Any adjustment in the CUB of genes associated with virulence will trigger expression and ultimately affect pathogenicity.

Colonisation in the host is mediated by the efficacy of degrading enzymes, which are a function of secretory proteins regulated by codon optimisation [42]. Any abnormality in these proteins negatively affects the host’s colonisation ability [95]. Thus, codon optimisation under the influence of translational selection regulates the colonisation and infectivity level of virulence factors in homogeneous and heterogenous host systems. Thus, understanding the CUB pattern of *Zn(II)_2_Cys_6_* would help to understand its role in causing plant infection. Furthermore, collaborating codon optimisation with modern day synthetic biology will add a feather to the cap. Codon optimisation gives an idea of an ideal gene that should function/express with a set of rules and regulations. At the same time, synthetic biology provides the platform to design the ideal gene. Synthetic biology is based on the concept of design-build-test-repeat, and bio-bricks form the basis of it [96,97,98]. The development of semi-synthetic artemisinin has been considered a breakthrough in the production of a potent anti-malarial drug [96]. *Zn(II)_2_Cys_6_* gene codons serve as parts; any modulation and re-arrangement in it will affect amino acids (device), which will lead to the rewiring of the genetic circuit (system), and ultimately impact the organism. Synthetic biology has already been applied to the production of amino acids (glutamic acid, lysine, methionine, lysine, etc.), using different chassis organisms [99]. Recently, by adding extra copies of six tRNA genes corresponding to *E. coli* CGG, GGA, CUA, CCC, AGA/AGG, and AUA minor codons in the BL21 strain of *E. coli* resulted in increased growth rate and higher expression of potential genes, subsequently enhancing translation rate in comparison with other non-modified strains [100]. Pathogens and hosts employ amino acids, which are biosynthetically cost-effective so that the saved energy can be channelized to impart more virulence or resistance to the system. Substitution of codons for amino acids, which would help to lower virulence in pathogens or increase resistance in plants, can be mediated to achieve disease-free plants. Synthetic genes based on codon optimisation parameters of a pathogen can be developed, which may help in imparting resistance to the plant. Alternately artificial genes may be constructed, which could impair the virulence of pathogens.

## 5. Conclusions

The present study was attempted to understand the codon usage pattern of the important transcription factor coding the *Zn(II)_2_Cys_6_* family, which is unique to fungi. The current study is the first of its kind, where a fungal-specific *Zn(II)_2_Cys_6_* gene family has been studied for codon usage bias among plant pathogenic Ascomycetes species in relation to model fungi yeast. The current investigation showed the influence of codon usage bias and nucleotide composition on the divergence of the *Zn(II)_2_Cys_6_* family between Ascomycetes and the model yeast system and within Ascomycetes species. Further, we found a combined influence of mutation pressure and natural and translational selections on codon usage bias of the *Zn(II)_2_Cys_6_* family. The study also identified common higher-represented codons specific to Ascomycetes and model fungi *S. cerevisiae*. The CUB of *Zn(II)_2_Cys_6_* sequences are directly relevant to the expression levels of genes. The preferable codons present in the genes could be targeted to decipher the molecular basis of the infection process and host–pathogen interactions through gene editing or knockouts.

## Figures and Tables

**Table 1:** The number of *Zn(II)_2_Cys_6_* coding sequences and codons considered for codon usage bias in Ascomycetes fungi and model *S. cerevisiae*.

Species	No. of CDS	No. of Codons
*Alternaria alternata*	133	92,890
*Aspergillus flavus*	348	221,472
*Bipolaris maydis*	192	129,680
*Bipolaris oryzae*	177	123,449
*Colletotrichum graminicola*	193	131,246
*Fusarium graminearum*	273	185,149
*Gaeumannomyces tritici*	152	111,329
*Pyricularia oryzae*	158	119,317
*Saccharomyces cerevisiae*	55	44,881
*Verticillium dahliae*	135	88,093

**Table 2:** The mean nucleotide composition of zinc binuclear cluster family CDS sequences in Ascomycetes plant pathogenic fungi and model yeast systems.

Fungi	%A	%T	%C	%G	%A3	%T3	%C3	%G3	%GC3	%GC
*Alternaria alternata*	25.39	21.99	28.10	24.52	21.58	23.23	29.93	25.26	55.19	52.52
*Aspergillus flavus*	24.60	23.80	26.91	24.69	21.16	25.44	27.50	25.90	53.40	51.66
*Bipolaris maydis*	25.35	22.33	28.43	23.89	21.80	23.41	30.55	24.24	54.78	52.21
*Bipolaris oryzae*	25.30	22.05	28.67	23.98	21.51	22.87	31.33	24.29	55.62	52.60
*Colletotrichum graminicola*	21.91	19.52	31.44	27.13	14.08	16.25	38.84	30.83	69.67	58.40
*Fusarium graminearum*	25.75	23.99	26.52	23.74	22.55	26.58	27.48	23.39	50.87	50.18
*Gaeumannomyces tritici*	19.66	17.10	33.85	29.39	10.60	12.23	42.80	34.37	77.17	63.49
*Pyricularia oryzae*	22.56	19.40	30.35	27.69	15.61	17.71	35.51	31.17	66.68	58.22
*Saccharomyces cerevisiae*	33.17	28.39	19.37	19.07	29.02	32.94	18.97	19.07	38.05	38.62
*Verticillium dahliae*	20.98	18.94	32.58	27.5	12.32	16.25	40.12	31.31	71.43	60.15

**Table 3:** Number of A/T- and G/C-rich overrepresented, underrepresented, less frequently used, and more frequently used codons.

Species	A/T				G/C			
	<0.6	0.6–1.0	1–1.60	>1.60	<0.6	0.6–1.0	1–1.60	>1.60
*Alternaria alternata*	2	19	9	-	-	10	18	1
*Aspergillus flavus*	0	18	12	-	-	10	19	0
*Bipolaris maydis*	1	20	9	-	1	8	20	1
*Bipolaris oryzae*	1	21	8	-	1	6	21	1
*Colletotrichum graminicola*	8	22	-	-	-	4	19	6
*Fusarium graminearum*	1	14	14	-	-	14	16	-
*Gaeumannomyces tritici*	23	7	-	-	1	1	17	10
*Pyricularia oryzae*	7	22	-	-	-	3	22	5
*Saccharomyces cerevisiae*	1	4	19	6	4	23	2	-
*Verticillium dahliae*	14	16	-	-	-	4	18	7

**Table 4:** The mean and range of translational selection indices for *zinc binuclear cluster* gene family coding sequences in Ascomycetes fungi and model yeast species.

Species	Minimum	Maximum	Mean
*Alternaria alternata*	0.39	0.65	0.55
*Aspergillus flavus*	0.30	0.70	0.50
*Bipolaris maydis*	0.31	0.68	0.50
*Bipolaris oryzae*	0.35	0.67	0.50
*Colletotrichum graminicola*	0.27	0.63	0.48
*Fusarium graminearum*	0.36	0.64	0.53
*Gaeumannomyces tritici*	0.24	0.60	0.41
*Pyricularia oryzae*	0.27	0.60	0.46
*Saccharomyces cerevisiae*	0.33	0.54	0.44
*Verticillium dahliae*	0.25	0.57	0.44

## Data Availability

All raw data included in the present investigation were mined from public databases. All analysed and supporting data is given in Supplementary File.

## Supplementary Materials

The following supporting information can be downloaded at: https://www.mdpi.com/article/10.3390/jof8111134/s1, Table S1: The composition and various codon usage bias parameters of 133 *Zn(II)_2_Cys_6_* sequences from *A. alternata*; Table S2: The composition and various codon usage bias parameters of 348 *Zn(II)_2_Cys_6_* sequences from *A. flavus*; Table S3: The composition and various codon usage bias parameters of 192 *Zn(II)_2_Cys_6_* sequences from *B. maydis*; Table S4: The composition and various codon usage bias parameters of 177 *Zn(II)_2_Cys_6_* sequences from *B. oryzae*; Table S5: The composition and various codon usage bias parameters of 193 *Zn(II)_2_Cys_6_* sequences from *C. graminicola*; Table S6: The composition and various codon usage bias parameters of 273 *Zn(II)_2_Cys_6_* sequences from *F. graminearum*; Table S7: The composition and various codon usage bias parameters of 152 *Zn(II)_2_Cys_6_* sequences from *G. tritici*; Table S8: The composition and various codon usage bias parameters of 158 *Zn(II)2Cys6* sequences from *P. oryzae*; Table S9: The composition and various codon usage bias parameters of 55 *Zn(II)_2_Cys_6_* sequences from *S. cerevisiae*; Table S10: The composition and various codon usage bias parameters of 135 *Zn(II)_2_Cys_6_* sequences from *V. dahliae*; Table S11: Relative synonymous codon usage bias analyses of *Zn(II)_2_Cys_6_* sequences from all ten target fungal species with emphasis on overrepresented and more frequently used codons; Table S12: The number and percentage of *Zn(II)_2_Cys_6_* sequences from all ten target species showing translational selection index greater or lesser than 0.50; Table S13: The percentage of variations explained by axis 1 and axis 2 from principal component analysis of RSCU of *Zn(II)_2_Cys_6_* sequences in target fungal species; Table S14: The correlation coefficients among the codon bias parameters and important compositional parameters of *Zn(II)_2_Cys_6_* sequences from *A. alternata;* Table S15: The correlation coefficients among the codon bias parameters and important compositional parameters of *Zn(II)_2_Cys_6_* sequences from *A. flavus;* Table S16: The correlation coefficients among the codon bias parameters and important compositional parameters of *Zn(II)_2_Cys_6_* sequences from *B. maydis;* Table S17: The correlation coefficients among the codon bias parameters and important compositional parameters of *Zn(II)_2_Cys_6_* sequences from *B. oryzae;* Table S18: The correlation coefficients among the codon bias parameters and important compositional parameters of *Zn(II)_2_Cys_6_* sequences from *C. graminicola;* Table S19: The correlation coefficients among the codon bias parameters and important compositional parameters of *Zn(II)_2_Cys_6_* sequences from *F. graminearum;* Table S20: The correlation coefficients among the codon bias parameters and important compositional parameters of *Zn(II)_2_Cys_6_* sequences from *G. tritici;* Table S21: The correlation coefficients among the codon bias parameters and important compositional parameters of *Zn(II)_2_Cys_6_* sequences from *P. oryzae;* Table S22: The correlation coefficients among the codon bias parameters and important compositional parameters of *Zn(II)_2_Cys_6_* sequences from *S. cerevesiae;* Table S23: The correlation coefficients among the codon bias parameters and important compositional parameters of *Zn(II)_2_Cys_6_* sequences from *V. dahliae*.

## References

[1] Schillig R., Morschhäuser J. (2013). Analysis of a fungus-specific transcription factor family, the *Candida albicans* zinc cluster proteins, by artificial activation. Mol. Microbiol..

[2] Atkinson T.J., Halfon M.S. (2014). Regulation of gene expression in the genomic context. Comput. Struct. Biotechnol. J..

[3] Shelest E. (2017). Transcription factors in fungi: TFome dynamics, three major families, and dual-specificity TFs. Front. Genet..

[4] MacPherson S., Larochelle M., Turcotte B. (2006). A fungal family of transcriptional regulators: The zinc cluster proteins. Microbiol. Mol. Biol. Rev..

[5] Zhang C., Huang H., Deng W., Li T. (2019). Genome-wide analysis of the Zn (II) 2Cys6 zinc cluster-encoding gene family in *Tolypocladium guangdongense* and its light-induced expression. Genes.

[6] Chang P.K., Ehrlich K.C. (2013). Genome-wide analysis of the Zn (II) 2Cys6 zinc cluster-encoding gene family in *Aspergillus flavus*. Appl. Microbiol. Biotechnol..

[7] Akache B., Wu K., Turcotte B. (2001). Phenotypic analysis of genes encoding yeast zinc cluster proteins. Nucleic Acids Res..

[8] Silver P.M., Oliver B.G., White T.C. (2004). Role of *Candida albicans* transcription factor Upc2p in drug resistance and sterol metabolism. Eukaryot. Cell..

[9] Schumacher D.I., Lütkenhaus R., Altegoer F., Teichert I., Kück U., Nowrousian M. (2018). The transcription factor PRO44 and the histone chaperone ASF1 regulate distinct aspects of multicellular development in the filamentous fungus *Sordaria macrospora*. BMC Genet..

[10] Hou Z., Chen Q., Zhao M., Huang C., Wu X. (2020). Genome-wide characterization of the Zn (II) 2Cys6 zinc cluster-encoding gene family in *Pleurotus ostreatus* and expression analyses of this family during developmental stages and under heat stress. PeerJ..

[11] Galhano R., Illana A., Ryder L.S., Rodriguez-Romero J., Demuez M., Badaruddin M., Sesma A. (2017). Tpc1 is an important Zn (II) 2Cys6 transcriptional regulator required for polarized growth and virulence in the rice blast fungus. PLoS Pathog..

[12] Marui J., Yamane N., Ohashi-Kunihiro S., Ando T., Terabayashi Y., Sano M., Machida M. (2011). Kojic acid biosynthesis in *Aspergillus oryzae* is regulated by a Zn (II) 2Cys6 transcriptional activator and induced by kojic acid at the transcriptional level. J. Biosci. Bioeng..

[13] Ammar H.A., Srour A.Y., Ezzat S.M., Hoseny A.M. (2017). Identification and characterization of genes involved in kojic acid biosynthesis in *Aspergillus flavus*. Ann. Microbiol..

[14] Seo J.A., Guan Y., Yu J.H. (2006). FluG-dependent asexual development in *Aspergillus nidulans* occurs via derepression. Genetics.

[15] Son Y.E., Cho H.J., Lee M.K., Park H.S. (2020). Characterizing the role of Zn cluster family transcription factor ZcfA in governing development in two *Aspergillus* species. PLoS ONE.

[16] Banerjee M., Thompson D.S., Lazzell A., Carlisle P.L., Pierce C., Monteagudo C., Kadosh D. (2008). UME6, a novel filament-specific regulator of *Candida albicans* hyphal extension and virulence. Mol. Biol. Cell..

[17] Tsuji G., Kenmochi Y., Takano Y., Sweigard J., Farrall L., Furusawa I., Kubo Y. (2000). Novel fungal transcriptional activators, Cmr1p of *Colletotrichum lagenarium* and Pig1p of *Magnaporthe grisea*, contain Cys2His2 zinc finger and Zn (II) 2Cys6 binuclear cluster DNA-binding motifs and regulate transcription of melanin biosynthesis genes in a developmentally specific manner. Mol. Microbiol..

[18] He Z., Dong Z., Qin L., Gan H. (2021). Phylodynamics and codon usage pattern analysis of broad bean wilt virus 2. Viruses.

[19] Hershberg R., Petrov D.A. (2008). Selection on Codon Bias. Annu. Rev. Genet..

[20] Labella A.L., Opulente D.A., Steenwyk J.L., Hittinger C.T., Rokas A. (2019). Variation and selection on codon usage bias across an entire subphylum. PLoS Genet..

[21] Wint R., Salamov A., Grigoriev I.V. (2022). Kingdom-Wide Analysis of Fungal Protein-Coding and tRNA Genes Reveals Conserved Patterns of Adaptive Evolution. Mol. Biol. Evol..

[22] Zhao F., Zhou Z., Dang Y., Na H., Adam C., Lipzen A., Liu Y. (2021). Genome-wide role of codon usage on transcription and identification of potential regulators. Proc. Natl. Acad. Sci. USA.

[23] Presnyak V., Alhusaini N., Chen Y.H., Martin S., Morris N., Kline N., Coller J. (2015). Codon optimality is a major determinant of mRNA stability. Cell..

[24] Stoletzki N., Eyre-Walker A. (2007). Synonymous codon usage in *Escherichia coli*: Selection for translational accuracy. Mol. Biol. Evol..

[25] Chevance F.F., Le Guyon S., Hughes K.T. (2014). The effects of codon context on *in vivo* translation speed. PLoS Genet..

[26] Buhr F., Jha S., Thommen M., Mittelstaet J., Kutz F., Schwalbe H., Komar A.A. (2016). Synonymous codons direct cotranslational folding toward different protein conformations. Mol. Cell..

[27] Zhou Z., Dang Y., Zhou M., Yuan H., Liu Y. (2018). Codon usage biases co-evolve with transcription termination machinery to suppress premature cleavage and polyadenylation. Elife.

[28] Deb B., Uddin A., Chakraborty S. (2021). Genome-wide analysis of codon usage pattern in herpesviruses and its relation to evolution. Virus Res..

[29] Ohama T., Muto A., Osawa S. (1990). Role of GC-biased mutation pressure on synonymous codon choice in *Micrococcus luteus* a bacterium with a high genomic GC-content. Nucleic Acids Res..

[30] Andersson S.G., Zomorodipour A., Andersson J.O., Sicheritz-Pontén T., Alsmark U.C., Podowski R.M., Näslund A.K., Eriksson A.S., Winkler H.H., Kurland C.G. (1998). The genome sequence of *Rickettsia prowazekii* and the origin of mitochondria. Nature.

[31] McInerney J.O. (1998). Replicational and transcriptional selection on codon usage in *Borrelia burgdorferi*. Proc. Natl. Acad. Sci. USA.

[32] Ikemura T. (1981). Correlation between the abundance of *Escherichia coli* transfer RNAs and the occurrence of the respective codons in its protein genes. J. Mol. Biol..

[33] De Miranda A.B., Alvarez-Valin F., Jabbari K., Degrave W.M., Bernardi G. (2000). Gene expression, amino acid conservation, and hydrophobicity are the main factors shaping codon preferences in *Mycobacterium tuberculosis* and *Mycobacterium leprae*. J. Mol. Evol..

[34] Powell J.R., Moriyama E.N. (1997). Evolution of codon usage bias in *Drosophila*. Proc. Natl. Acad. Sci. USA.

[35] Duret L., Marais G., Biémont C. (2000). Transposons but not retrotransposons are located preferentially in regions of high recombination rate in *Caenorhabditis elegans*. Genetics.

[36] Chen Y. (2013). A comparison of synonymous codon usage bias patterns in DNA and RNA virus genomes: Quantifying the relative importance of mutational pressure and natural selection. BioMed. Res. Int..

[37] Frenkel Morgenstern M., Danon T., Christian T., Igarashi T., Cohen L., Hou Y.M., Jensen L.J. (2012). Genes adopt non-optimal codon usage to generate cell cycle-dependent oscillations in protein levels. Mol. Syst. Biol..

[38] Barbhuiya P.A., Uddin A., Chakraborty S. (2019). Compositional properties and codon usage of TP73 gene family. Gene.

[39] Xu Y., Ma P., Shah P., Rokas A., Liu Y., Johnson C.H. (2013). Non-optimal codon usage is a mechanism to achieve circadian clock conditionality. Nature.

[40] Arella D., Dilucca M., Giansanti A. (2021). Codon usage bias and environmental adaptation in microbial organisms. Mol. Genet. Genom..

[41] Adams M.J., Antoniw J.F. (2003). Codon usage bias amongst plant viruses. Arch Virol..

[42] Badet T., Peyraud R., Mbengue M., Navaud O., Derbyshire M., Oliver R.P., Barbacci A., Raffaele S. (2017). Codon optimization underpins generalist parasitism in fungi. Elife.

[43] Duret L., Mouchiroud D. (1999). Expression pattern and, surprisingly, gene length shape codon usage in *Caenorhabditis*, *Drosophila*, and *Arabidopsi*s. Proc. Natl. Acad. Sci. USA.

[44] Roymondal U., Das S., Sahoo S. (2009). Predicting gene expression level from relative codon usage bias: An application to *Escherichia coli* genome. DNA Res..

[45] Yang X., Luo X., Cai X. (2014). Analysis of codon usage pattern in *Taenia saginata* based on a transcriptome dataset. Parasites Vectors.

[46] Roy A., van Staden J. (2019). Comprehensive profiling of codon usage signatures and codon context variations in the genus *Ustilago*. World J. Microbiol. Biotechnol..

[47] Dean R., Van Kan J.A., Pretorius Z.A., HammonduKosack K.E., Di Pietro A., Spanu P.D., Rudd J.J., Dickman M., Kahmann R., Ellis J. (2012). The Top 10 fungal pathogens in molecular plant pathology. Mol. Plant Pathol..

[48] Li J., Gu F., Wu R., Yang J.K., Zhang K.Q. (2017). Phylogenomic evolutionary surveys of subtilase superfamily genes in fungi. Sci. Rep..

[49] Nalley L., Tsiboe F., Durand-Morat A., Shew A., Thoma G. (2016). Economic and environmental impact of rice blast pathogen (*Magnaporthe oryzae*) alleviation in the United States. PLoS ONE.

[50] De Lucca A.J. (2007). Harmful fungi in both agriculture and medicine. Rev. Iberoam Micol..

[51] Vesonder R., Haliburton J., Stubblefield R., Gilmore W., Peterson S. (1991). *Aspergillus flavus* and aflatoxins B1, B2, and M1 in corn associated with equine death. Arch. Environ. Contam. Toxicol..

[52] Pitt J.I. (2000). Toxigenic fungi: Which are important?. Sabouraudia..

[53] Navale V., Vamkudoth K.R., Ajmera S., Dhuri V. (2021). *Aspergillus* derived mycotoxins in food and the environment: Prevalence, detection, and toxicity. Toxicol. Rep..

[54] Song H., Liu J., Song Q., Zhang Q., Tian P., Nan Z. (2017). Comprehensive analysis of codon usage bias in seven *Epichloë* species and their peramine-coding genes. Front. Microbiol..

[55] Wright F. (1990). The effective number of codons' used in a gene. Gene.

[56] Sharp P.M., Li W.H. (1986). An evolutionary perspective on synonymous codon usage in unicellular organisms. J. Mol. Evol..

[57] Shields D.C., Sharp P.M., Higgins D.G., Wright F. (1988). Silent" sites in Drosophila genes are not neutral: Evidence of selection among synonymous codons. Mol. Biol. Evol..

[58] Freire-Picos M.A., Gonzalez-Siso M.I., Rodríguez-Belmonte E., Rodríguez-Torres A.M., Ramil E., Cerdan M.E. (1994). Codon usage in *Kluyveromyces lactis* and in yeast cytochrome c-encoding genes. Gene.

[59] Gatherer D., McEwan N.R. (1997). Small regions of preferential codon usage and their effect on overall codon bias-The case of the plp gene. IUBMB Life.

[60] Gustafsson C., Minshull J., Govindarajan S., Ness J., Villalobos A., Welch M. (2012). Engineering genes for predictable protein expression. Protein Expr. Purifi..

[61] Choudhury M.N., Uddin A., Chakraborty S. (2017). Codon usage bias and its influencing factors for Y-linked genes in human. Comput. Biol. Chem..

[62] Bahiri-Elitzur S., Tuller T. (2021). Codon-based indices for modeling gene expression and transcript evolution. Comput. Struct. Biotechnol. J..

[63] Bourret J., Alizon S., Bravo I.G. (2008). COUSIN (COdon Usage Similarity INdex): A normalized measure of codon usage preferences. Genome Biol. Evol..

[64] Kyte J., Doolittle R.F. (1982). A simple method for displaying the hydropathic character of a protein. J. Mol. Biol..

[65] Lobry J.R., Gautier C. (1999). Hydrophobicity, expressivity and aromaticity are the major trends of amino-acid usage in 999 *Escherichia coli* chromosome-encoded genes. Nucleic Acids Res..

[66] Sueoka N. (2001). Near homogeneity of PR2-bias fingerprints in the human genome and their implications in phylogenetic analyses. J. Mol. Evol..

[67] Sueoka N. (1995). Intrastrand parity rules of DNA base composition and usage biases of synonymous codons. J. Mol. Evol..

[68] Zhang W.J., Zhou J., Li Z.F., Wang L., Gu X., Zhong Y. (2007). Comparative analysis of codon usage patterns among mitochondrion, chloroplast and nuclear genes in *Triticum aestivum L*. J. Integr. Plant Biol..

[69] Wu Y., Zhao D., Tao J. (2015). Analysis of codon usage patterns in herbaceous peony (*Paeonia lactiflora* Pall.) based on transcriptome data. Genes.

[70] Gouy M., Gautier C. (1982). Codon usage in bacteria: Correlation with gene expressivity. Nucleic Acids Res..

[71] Butt A.M., Nasrullah I., Qamar R., Tong Y. (2016). Evolution of codon usage in Zika virus genomes is host and vector specific. Emerg. Microbes Infect..

[72] Wang L., Xing H., Yuan Y., Wang X., Saeed M., Tao J., Sun X. (2018). Genome-wide analysis of codon usage bias in four sequenced cotton species. PLoS ONE.

[73] Liu Q. (2012). Mutational bias and translational selection shaping the codon usage pattern of tissue-specific genes in rice. PLoS ONE.

[74] Li X., Song H., Kuang Y., Chen S., Tian P., Li C., Nan Z. (2017). Genome-wide analysis of codon usage bias in *Epichloe festucae*. Int. J.Mol.Sci..

[75] Chandan J., Gupta S., Babu V., Singh D., Singh R. (2022). Comprehensive analysis of codon usage pattern in *Withania somnifera* and its associated pathogens: *Meloidogyne incognita* and *Alternaria alternata*. Genetica.

[76] Kawabe A., Miyashita N.T. (2003). Patterns of codon usage bias in three dicot and four monocot plant species. Genes Genet. Syst..

[77] Carbone A., Zinovyev A., Képes F. (2003). Codon adaptation index as a measure of dominating codon bias. Bioinform..

[78] Puigbò P., Bravo I.G., Garcia-Vallve S. (2008). CAIcal: A combined set of tools to assess codon usage adaptation. Biol. Direct..

[79] Gupta S., Singh R. (2021). Comparative study of codon usage profiles of *Zingiber officinale* and its associated fungal pathogens. Mol. Genet. Genom..

[80] Jenkins G.M., Holmes E.C. (2003). The extent of codon usage bias in human RNA viruses and its evolutionary origin. Virus Res..

[81] Parvathy S.T., Udayasuriyan V., Bhadana V. (2022). Codon usage bias. Mol. Biol. Rep..

[82] Plotkin J.B., Kudla G. (2011). Synonymous but not the same: The causes and consequences of codon bias. Nature Rev. Genet..

[83] Uddin A., Choudhury M.N., Chakraborty S. (2017). Factors influencing codon usage of mitochondrial ND1 gene in pisces, aves and mammals. Mitochondrion..

[84] Das S., Uddin A., Bhattacharyya D., Chakraborty S. (2019). Transcript free energy positively correlates with codon usage bias in mitochondrial genes of *Calypogeia* species (*Calypogeiaceae*, *Marchantiophyta*). Mitochondrial DNA A DNA Mapp Seq Anal..

[85] Jiang W., Lv B., Wu X., Wang J., Wu G., Shi C., Tang X. (2017). Analysis of synonymous codon usage patterns in the edible fungus *Volvariella volvacea*. Biotechnol. Appl. Biochem..

[86] Malakar A.K., Halder B., Paul P., Chakraborty S. (2016). Cytochrome P450 genes in coronary artery diseases: Codon usage analysis reveals genomic GC adaptation. Gene.

[87] Liu H., Huang Y., Du X., Chen Z., Zeng X., Chen Y., Zhang H. (2012). Patterns of synonymous codon usage bias in the model grass *Brachypodium distachyon*. Genet Mol. Res..

[88] Athey J., Alexaki A., Osipova E., Rostovtsev A., Santana-Quintero L.V., Katneni U., Simonyan V., Kimchi-Sarfaty C. (2017). A new and updated resource for codon usage tables. BMC Bioinform..

[89] Suzuki N., Supyani S., Maruyama K., Hillman B.I. (2004). Complete genome sequence of Mycoreovirus-1/Cp9B21, a member of a novel genus within the family Reoviridae, isolated from the chestnut blight fungus *Cryphonectria parasitica*. J. Gen. Virol..

[90] Lucks J.B., Nelson D.R., Kudla G.R., Plotkin J.B. (2008). Genome landscapes and bacteriophage codon usage. PLoS Comput. Biol..

[91] Bahir I., Fromer M., Prat Y., Linial M. (2009). Viral adaptation to host: A proteome-based analysis of codon usage and amino acid preferences. Mol. Syst. Biol..

[92] Biswas K., Palchoudhury S., Chakraborty P., Bhattacharyya U.K., Ghosh D.K., Debnath P., Lee R.F. (2019). Codon usage bias analysis of *citrus tristeza* virus: Higher codon adaptation to *Citrus reticulate* host. Viruses.

[93] Sudha S.N., Krishnaswamy S., Sekar V. (1992). Comparison of codon usage in genes of plant viruses and their hosts. Curr. Sci..

[94] Nisa S., Gupta S., Ahmed W., Singh R. (2022). Deciphering the role of codon usage bias on gene expression and pathogen colonization in Crocus sativus. Res. Sq..

[95] Peyraud R., Cottret L., Marmiesse L., Gouzy J., Genin S. (2016). A resource allocation trade-off between virulence and proliferation drives metabolic versatility in the plant pathogen *Ralstonia solanacearum*. PLoS Pathog..

[96] Gibson D.G., Glass J.I., Lartigue C., Noskov V.N., Chuang R.Y., Algire M.A., Benders G.A., Montague M.G., Ma L., Moodie M.M. (2010). Creation of a bacterial cell controlled by a chemically synthesized genome. Science.

[97] Paddon C.J., Keasling J.D. (2014). Semi-synthetic artemisinin: A model for the use of synthetic biology in pharmaceutical development. Nat. Rev. Microbiol..

[98] El Karoui M., Hoyos-Flight M., Fletcher L. (2019). Future trends in synthetic biology—A report. Front. Bioeng. Biotechnol..

[99] Singh S., Tiwari B.S. (2019). Biosynthesis of high-value amino acids by synthetic biology. Curr. Dev. Biotechnol. Bioeng..

[100] Lipinszki Z., Vernyik V., Farago N., Sari T., Puskas L.G., Blattner F.R., Posfai G., Gyorfy Z. (2018). Enhancing the translational capacity of *E. coli* by resolving the codon bias. ACS Synth. Biol..

